# Photosystem II of *Ligustrum lucidum* in response to different levels of manganese exposure

**DOI:** 10.1038/s41598-019-48735-8

**Published:** 2019-08-29

**Authors:** Hui-Zi Liang, Fan Zhu, Ren-Jie Wang, Xin-Hao Huang, Jing-Jing Chu

**Affiliations:** 1 College of Life Science and Technology, Central South University of Forestry and Technology, Changsha, 410004 P. R. China; 2 Engineering Laboratory of Applied Technology for Forestry & Ecology in Southern China, Changsha, 410004 Hunan China

**Keywords:** Photosystem II, Abiotic

## Abstract

The toxic effect of excessive manganese (Mn) on photosystem II (PSII) of woody species remains largely unexplored. In this study, five Mn concentrations (0, 12, 24, 36, and 48 mM) were used, and the toxicity of Mn on PSII behavior in leaves of *Ligustrum lucidum* was investigated using *in vivo* chlorophyll fluorescence transients. Results showed that excessive Mn levels induced positive L- and K- bands. Variable fluorescence at 2 ms (V_J_) and 30 ms (V_I_), absorption flux (ABS/RC), trapped energy flux (TR_o_/RC), and dissipated energy flux (DI_o_/RC) increased in Mn-treated leaves, whereas the performance index (PI_ABS_), electron transport flux (ET_o_/RC), maximum quantum yield (φ_Po_), quantum yield of electron transport (φ_Eo_), and probability that an electron moves further than Q_A_^−^ (ψ_o_) decreased. Also, excessive Mn significantly decreased the net photosynthesis rate and increased intercellular CO_2_ concentration. The results indicated that Mn blocked the electron transfer from the donor side to the acceptor side in PSII, which might be associated with the accumulation of Q_A_^−^, hence limiting the net photosynthetic rate.

## Introduction

It is well-known that manganese (Mn) is an essential micronutrient element required for the growth and development of plants. Especially, Mn is involved in metabolic pathways of chlorophyll (Chl) synthesis and breakdown in the chloroplasts^1,2^. The oxygen-evolving complex (OEC) of photosystem II (PSII) contains a Mn-containing metalloenzyme core. This inorganic core, binding to the reaction center (RC) protein D1 in PSII, has the empirical formula Mn_4_CaO_5_ and is known as the tetra-nuclear Mn cluster^3,4^. However, Mn, in excess, is also considered as one of the most toxic trace metals to plants. Mn pollution often originates from industrial disposal^5^, manufacturing sewage^6,7^, as well as mineral exploitation^8^.

Toxic effects of Mn on plants is well documented. Excessive Mn level impedes plant growth and development by interfering with metabolic processes^9,10^. Moreover, a number of studies have shown that Mn mainly exerts its toxicity to plant leaves by inhibiting photosynthesis and chloroplast activity^9–12^ leading to reduced chloroplast content and suppressed CO_2_ assimilation^13–16^. PSII is highly sensitive to Mn level. Feng *et al*.^13^ have reported that excessive Mn inhibits the maximum photochemical efficiency (F_v_/F_M_) and effective quantum yield of PSII (ΦPSII) in cucumber. Doncheva *et al*.^17^ have shown that excessive Mn significantly affects the quantum efficiency of PSII in Mn-sensitive maize (*Zea mays L*.) ‘Kneja 605’, but not in Mn-tolerant maize ‘Kneja 434’. Interestingly, some other studies have revealed that F_v_/F_M_ is not substantially affected by Mn accumulation in tobacco and rice bean seedlings^11,18^. In addition, Kitao *et al*.^19^ have suggested that excessive Mn affects the activity of CO_2_ reduction cycle rather than F_v_/F_M_ in white birch, while increased Q_A_ reduction and thermal energy dissipation, as well as decreased quantum yield of PSII, have been observed. Similar results have been found in *Alnus hirsuta* Turcz., *Betula ermanii* Cham., *Ulmus davidiana* Planch., and *Acer mono* Maxim^15^. Li *et al*.^16^ have reported that excessive Mn impairs the whole photosynthetic electron transport chain from the donor side of PSII up to the reduction of end acceptors of PSI in *Citrus grandis* seedlings, followed by the increase in ABS/RC, TR_o_/RC, and DI_o_/RC, as well as decrease in ET_o_/RC, φ_Po_, and ψ_o_. However, the mechanism of toxicity of Mn to PSII remains largely unexplored, and most previous studies have only focused on herbaceous plants^11,13,17,18^.

Chl a fluorescence, a non-invasive spectroscopic technique, has been widely used to detect and measure the *in vivo* behavior of PSII under different environmental stresses^20,21^. Analysis of the polyphasic fluorescence transient under physiological conditions shows that the fluorescence increases in the typical shape of OJIP kinetics^21^. The “JIP-test” analysis of the OJIP transients allows the calculation of structural, conformational, and functional parameters quantifying the PSII behavior under environmental stresses, including absorption flux, trapped energy flux, electron transport flux, and dissipated energy flux^21^.

Hunan Province in the southern area of China has a high density of Mn mines. Severe pollution in agricultural lands, stream water, sediments, and soils have been reported in this area, threatening human health^8^. As an important evergreen broad-leaved tree species in the southern regions of China, *Ligustrum lucidum* is highly tolerant to heavy metals^22,23^. In this study, we aimed to investigate changes in the OJIP transient and related parameters in the leaves of *L*. *lucidum* in the presence of Mn. In addition, we evaluated the toxicity of Mn to PSII behavior when *L*. *lucidum* was cultured under Mn stress for up to 40 days.

## Materials and Methods

### Plant culture and Mn treatments

Two-year-old *L*. *lucidum* seedlings (average diameter, ~9 mm; height, ~133 cm) were purchased from a local nursery. All plants were individually transplanted into plastic pots (diameter, 25.4 cm; height, 17.8 cm) filled with 7 kg of air-dried soil. The plants were grown under natural illumination (30/25 °C day/night temperature, 12/12 h day/night cycle and a maximum photosynthetically active radiation of about 1,000 *μ*mol photons m^−2^ s^−1^) for 4 months to acclimatize them to the soil microclimate before initiating Mn treatment. Each pot was supplied with 400 mL of pure water every 2 to 3 days.

Samples of soil free from heavy metal pollution were collected from the CSUFT campus soil at a depth of 5–20 cm. The soil samples were taken back to the laboratory, and were sieved through 5 × 5 mm sieves to remove rocks, and were then air-dried at room temperature. The chemical properties of soil samples were as measured: pH 4.9, 0.227 g N/kg, 0.129 g P/kg, and 355.978 mg Mn/kg.

For the Mn-treated soil, distilled water containing 1.2 mM, 2.4 mM, 3.6 mM and 4.8 mM Mn from MnCl_2_·5H_2_O was added to the pots every other day at a rate of 400 mL per day for 20 days. The four Mn treatments were designated as L1, L2, L3, and L4, respectively. For the control (CK), about 400 mL of distilled water without Mn was added into the pots. In total we have five treatments: CK, L1, L2, L3 and L4, and each treatment was replicated five times. Measurements were carried out on three fully expanded *L*. *lucidum* leaves of similar size on days 10, 25, and 40 after the Mn treatment.

### Fast Chl a fluorescence kinetics and JIP-test

Fast Chl a fluorescence was measured by M-PEA (Multifunctional Plant Efficiency Analyzer, Hansatech Instrument, UK). Leaves were exposed to a pulse of saturating red light (5,000 *μ*mol m^−2^ s^−1^, peak 625 nm, duration 50 μs–2 s, records of 128 points) and measured daily between 8:30–11:00 am after 1 h of dark adaptation using dark adaptation clips. The fluorescence transients (OJIP curves) were analyzed to determine energy distribution through PSII per RC (ABS/RC, TR_o_/RC, ET_o_/RC, DI_o_/RC, see Table 1), flux ratios (φ_Po_, φ_Eo_, and ψ_o_) and performance index (PI_ABS_) according to the JIP-test^19^. Relative variable fluorescence at time t, at the J-step, and at the I-step (i.e., V_t_, V_J_ and V_I_, respectively) was calculated using the following equations^16,21^:

1 $${{\rm{V}}}_{{\rm{t}}}=({{\rm{F}}}_{{\rm{t}}}-{{\rm{F}}}_{{\rm{o}}})/({{\rm{F}}}_{{\rm{M}}}-{{\rm{F}}}_{{\rm{o}}})$$

2 $${{\rm{V}}}_{{\rm{J}}}=({{\rm{F}}}_{{\rm{J}}}-{{\rm{F}}}_{{\rm{o}}})/({{\rm{F}}}_{{\rm{M}}}-{{\rm{F}}}_{{\rm{o}}})$$

3 $${{\rm{V}}}_{{\rm{I}}}=({{\rm{F}}}_{{\rm{I}}}-{{\rm{F}}}_{{\rm{o}}})/({{\rm{F}}}_{{\rm{M}}}-{{\rm{F}}}_{{\rm{o}}})$$

4 $${{\rm{\Delta }}V}_{{\rm{t}}}={{\rm{V}}}_{{\rm{t}}}-{{\rm{V}}}_{{\rm{t}}({\rm{control}})}$$

where F_J_ is fluorescence intensity at 2 ms and F_I_ is the fluorescence intensity at 30 ms.

**Table 1:** Definition of terms and formulae of the selected JIP-test parameters.

**Data extracted from the recorded fluorescence transient OJIP**	
F_o_ $\cong$ F_50μs_	Minimal fluorescence, when all RCs are open
F_300μs_, F_2ms_, F_30ms_	Fluorescence intensity at 300 μs, 2 ms, 30 ms, respectively
**FPs derived from the extracted data**	
V_t_ = (F_t_ − F_o_)/(F_M_ − F_o_)	Relative variable fluorescence at time t
V_J_ = (F_2ms_ − F_o_)/(F_M_ − F_o_)	Relative variable fluorescence at the J-step (2 ms), reflects the activity of acceptor side of PSII
V_I_ = (F_30ms_ − F_o_)/(F_M_ − F_o_)	Relative variable fluorescence at the I-step (30 ms), reflects the activity of acceptor side of PSII
**Specific energy fluxes (per Q**_**A**_ **reducing PSII RC)**	
ABS/RC	Absorption flux (of antenna Chls) per RC (also a measure of PSII apparent antenna size)
TR_o_/RC	Trapped energy flux (leading to Q_A_ reduction) per RC at t = 0
ET_o_/RC	Electron transport flux (further than Q_A_^−^) per RC at t = 0
DI_o_/RC	Dissipated energy flux per RC at t = 0
**Quantum yields and efficiencies/probabilities**	
φ_Po_ = TR_o_/ABS	Maximum quantum yield for primary photochemistry
φ_Eo_ = ET_o_/ABS	Quantum yield for electron transport
Ψ_o_ = ET_o_/TR_o_	Efficiency/probability that an electron moves further than Q_A_^−^
**Performance index**	
PI_ABS_ = (RC/ABS)(φ_Po_/1 − φ_Po_))(ψ_o_/(1 − ψ_o_))	Performance index (potential) for energy conservation from photons absorbed by PSII to the reduction of intersystem electron acceptors

To further characterize the effect of Mn on *L*. *lucidum* PSII, some functional parameters were calculated from the JIP-test. The OJIP transients were double normalized between O (50 μs) and P steps to estimate relative variable fluorescence W_OP_ = (F_t_ − F_o_)/(F_P_ − F_o_). Normalization between O and K (300 μs) steps revealed L-band (150 μs), resulting in the variable fluorescence^24^:

5 $${{\rm{W}}}_{{\rm{OK}}}=({{\rm{F}}}_{{\rm{t}}}-{{\rm{F}}}_{{\rm{o}}})/({{\rm{F}}}_{{\rm{K}}}-{{\rm{F}}}_{{\rm{o}}})$$

6 $${{\rm{W}}}_{{\rm{OK}}({\rm{control}})}=({{\rm{F}}}_{{\rm{t}}}-{{\rm{F}}}_{{\rm{o}}})/({{\rm{F}}}_{{\rm{K}}}-{{\rm{F}}}_{{\rm{o}}})$$

7 $${{\rm{\Delta }}{\rm{W}}}_{{\rm{OK}}}={{\rm{W}}}_{{\rm{OK}}}-{({{\rm{W}}}_{{\rm{OK}}})}_{{\rm{control}}}$$

Normalization between O and J (2 ms) steps revealed K-band (300 μs), resulting in the variable fluorescence^24^:

8 $${{\rm{W}}}_{{\rm{OJ}}}=({{\rm{F}}}_{{\rm{t}}}-{{\rm{F}}}_{{\rm{o}}})/({{\rm{F}}}_{{\rm{J}}}-{{\rm{F}}}_{{\rm{o}}})$$

9 $${{\rm{\Delta }}{\rm{W}}}_{{\rm{OJ}}}={{\rm{W}}}_{{\rm{OJ}}}-{({{\rm{W}}}_{{\rm{OJ}}})}_{{\rm{control}}}$$

The F_I_, F_J_, F_K_, F_M_, and F_O_ represent fluorescence at I-step, J-step, and K-step, dark-adapted maximum fluorescence, and dark-adapted minimum fluorescence, respectively. ΔV_J_, ΔV_I_, ΔW_OK_, and ΔW_OJ_ represent the J-band, I-band, L-band, and K-band, respectively, and are associated with the accumulation of Q_A_^−^^21^, the proportion of Q_B_-non-reducing PSII RCs^16^, energetic connectivity of antennae to PSII RC units^24^, and the activity of OEC of PSII donor side^25^, respectively.

### Gas exchange

Net photosynthetic rate (Pn) and intercellular CO_2_ concentration (Ci) were measured by LI-COR 6400 portable photosynthesis system (LI-COR Bioscience, Lincoln, NE, USA). Measurements were carried out on three leaves per plant on day 40 with 1,000 μmol photon m^−2^ s^−1^, and a 2 min measurement duration per sample.

### Determination of total Mn content

The dried biomass of different organs (roots, stems, and leaves) was powdered and used to digested with 15 mL acid mixture (HClO_4_/HNO_3_ = 1/4). The concentration of Mn was determined by ICP-AES (Optima 8300, American platinum Elmer, USA).

### Data analysis

Data were reported as means of each group based on at least six independent replicates. Results are presented as means ± standard error (SE). Statistical differences between measurements were analyzed using one-way ANOVA, followed by a least significant difference (LSD) test at *P* < 0.05. Chl a fluorescence parameters (FPs) associated with Mn levels and stress time were assessed using two-way ANOVA with α = 0.05. All graphs were made using Sigmaplot 12.0.

## Results

### Effects of Mn on Chl a fluorescence transient and related parameters in *L*. *lucidum* leaves

**Figure 1:**
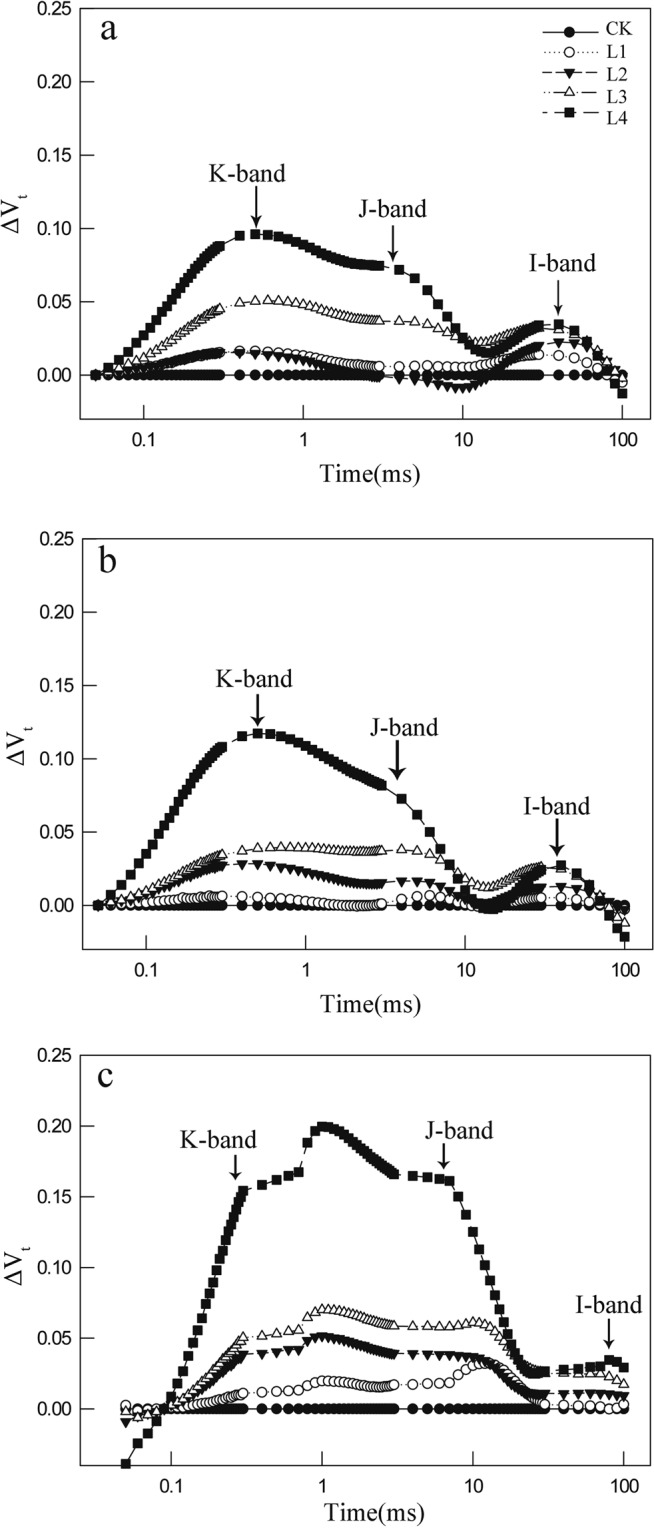
Changes in O-P phase relative variable fluorescence intensity (ΔV_t_) in control group and four Mn-treated groups on day 10 (**a**), day 25 (**b**) and day 40 (**c**).

All OJIP transients from both Mn-treated and control leaves showed a typical polyphasic rise with the basic steps of O-J-I-P. Mn-treated leaves displayed positive trends of K-, J-, and I-bands compared with the controls at 300 μs, 2 ms, and 30 ms, respectively (Fig. 1). K, J, or I step was positively correlated with the Mn level or the stress time in the Mn-treated leaves.

**Figure 2:**
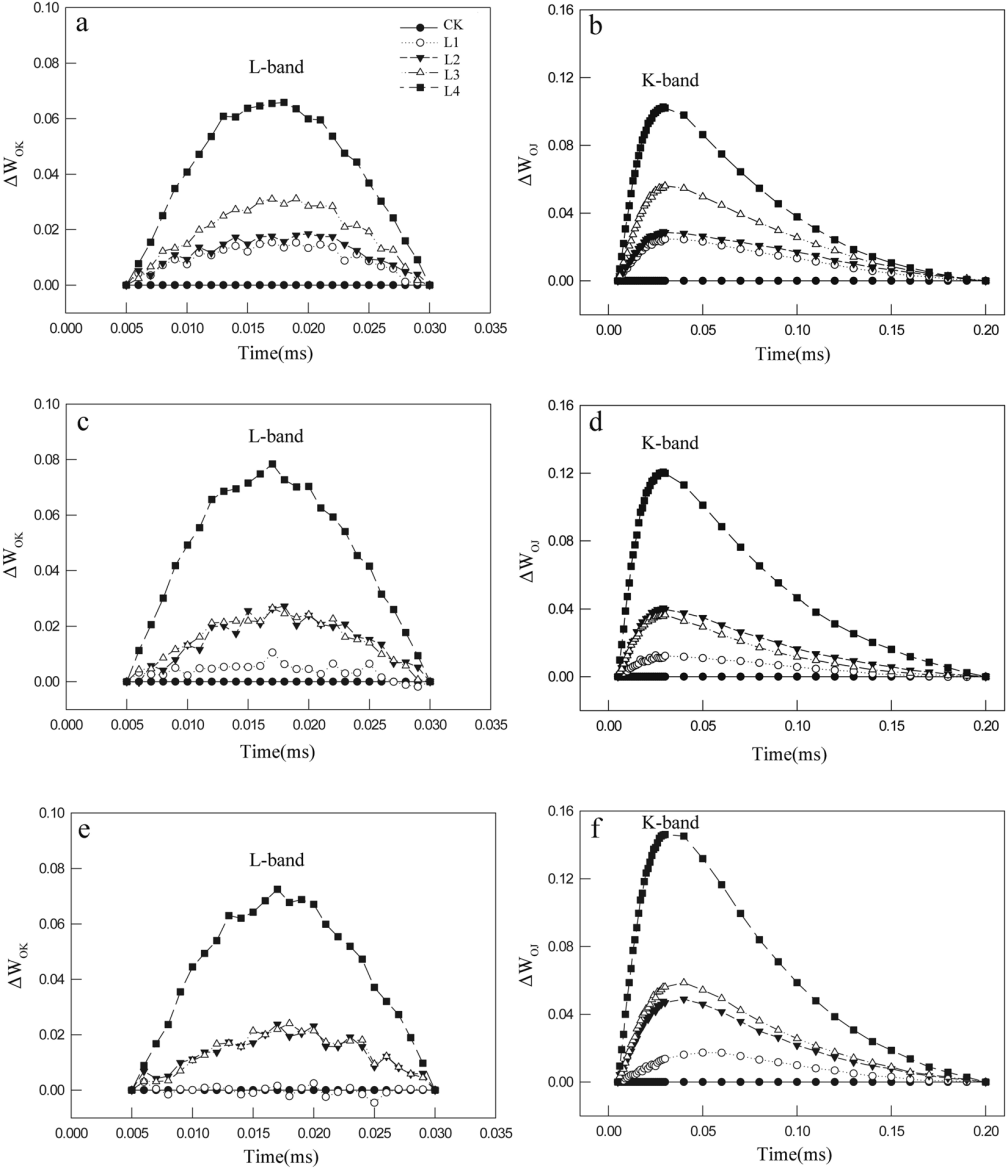
Changes in O-K phase relative variable fluorescence intensity (ΔW_OK_, **a**–**c**), and in O-J phase relative variable fluorescence intensity (ΔW_OJ_, **d**–**f**) in control group and four Mn-treated groups on day 10 (**a**,**d**), day 25 (**b**,**e**) and day 40 (**c**,**f**).

Positive L-band and K-band increased with the Mn levels on days 10, 25, and 40 (Fig. 2). In the L1-treated group, the L-band achieved the maximum value on day 10 and then decreased continuously to reach the control levels on day 40. In the L2-treated group, the maximum value of L-band appeared on day 25 and was maintained until day 40. The changes of K-band were similar to L-band except that the K-band in the L1-treated group on day 40 was higher than that of the controls.

Variable fluorescence at 2 ms (V_J_) increased with the Mn levels (Table 2). There was no significant difference between the L1- or the L2-treated groups and control group except for the L2-treated group on day 40. A significant difference was observed between the L3- and L4-treated groups and the control group. Significant differences were also observed between the L4-treated group and the L1- or L2-treated groups. V_J_ was significantly higher on day 40 compared with day 10 in all Mn treatment levels. The variable fluorescence at 30 ms (V_I_) increased with Mn levels (Table 2). V_I_ was significantly increased in L2-, L3- and L4-treated groups compared with the control group, while there was no significant difference between the L1-treated group and the control group. V_I_ was significantly increased in the L4-treated group compared with the L2-treated group on days 25 and day 40. There were no significant differences among the different stress time points.

**Table 2:** Fluorescence parameters (V_J_ and V_I_) in the control and Mn-treated groups on day 10, day 25 and day 40.

	Mn-treatment	Day 10	Day 25	Day 40
V_J_	CK	0.530 ± 0.009 Aa	0.537 ± 0.010 Aa	0.542 ± 0.022 Aa
	L1	0.539 ± 0.005 ABa	0.538 ± 0.008 Aa	0.557 ± 0.004 ABb
	L2	0.534 ± 0.010 ABa	0.557 ± 0.014 Aab	0.588 ± 0.015 BCb
	L3	0.572 ± 0.009 Ba	0.576 ± 0.004 ABab	0.606 ± 0.005 Cb
	L4	0.614 ± 0.029 Ca	0.638 ± 0.045 Bab	0.720 ± 0.017 Db
V_I_	CK	0.892 ± 0.005 Aa	0.896 ± 0.004 Aa	0.901 ± 0.004 Aa
	L1	0.905 ± 0.005ABa	0.902 ± 0.004 ABa	0.902 ± 0.004 ABa
	L2	0.912 ± 0.006 BCa	0.909 ± 0.004 BCa	0.913 ± 0.006 Ba
	L3	0.923 ± 0.005 Ca	0.922 ± 0.004 CDa	0.926 ± 0.001 Ca
	L4	0.926 ± 0.007 Ca	0.922 ± 0.003 Da	0.933 ± 0.002 Ca

Values represent mean ± SE. Different capital letters represent significant difference between the different Mn-treatments in the same stress time points, and different lowercase letters represent significant difference between the same Mn- treatments in the different stress time points (*P* < 0.05).

Mn-treatment = Manganese-treatment; CK = control; L1 = 12 mM manganese -treatment; L2 = 24 mM manganese -treatment; L3 = 36 mM manganese -treatment; L4 = 48 mM manganese -treatment; V_J_ = values of relative variable fluorescence at 2 ms; V_I_ = values of relative variable fluorescence at 30 ms.

**Table 3:** Two-way ANOVA results for JIP-test parameters.

	Mn level	Stress time	Mn level × Stress time
V_J_	27.176**	10.695**	1.519
V_I_	24.389**	1.396	0.427
PI_ABS_	27.454**	5.753**	0.571
ABS/RC	14.637**	2.039	0.367
TR_0_/RC	12.174**	1.802	0.279
ET_0_/RC	3.879**	7.108**	0.921
DI_0_/RC	17.893**	2.257	0.625
φ_PO_	36.393**	0.065	0.204
φ_EO_	31.667**	10.882**	1.515
ψ_0_	27.176**	10.695**	1.519

*P < 0.05, **P < 0.01.

V_J_ = values of relative variable fluorescence at 2 ms; V_I_ = values of relative variable fluorescence at 30 ms; PI_ABS_ = Performance index (potential) for energy conservation from photons absorbed by PSII to the reduction of intersystem electron acceptors; ABS/RC = Absorption flux (of antenna Chls) per RC (also a measure of PSII apparent antenna size); TR_o_/RC = Trapped energy flux (leading to Q_A_ reduction) per RC at t = 0; ET_o_/RC = Electron transport flux (further than Q_A_^−^) per RC at t = 0; DI_o_/RC = Dissipated energy flux per RC at t = 0; φ_Po_ = Maximum quantum yield for primary photochemistry; φ_Eo_ = Quantum yield for electron transport; ψ_o_ = Efficiency/probability that an electron moves further than Q_A_^−^.

### Effects of Mn on the performance index, energy distribution, and the quantum yield of excitation energy trapping of PSII in *L*. *lucidum* leaves

**Figure 3:**
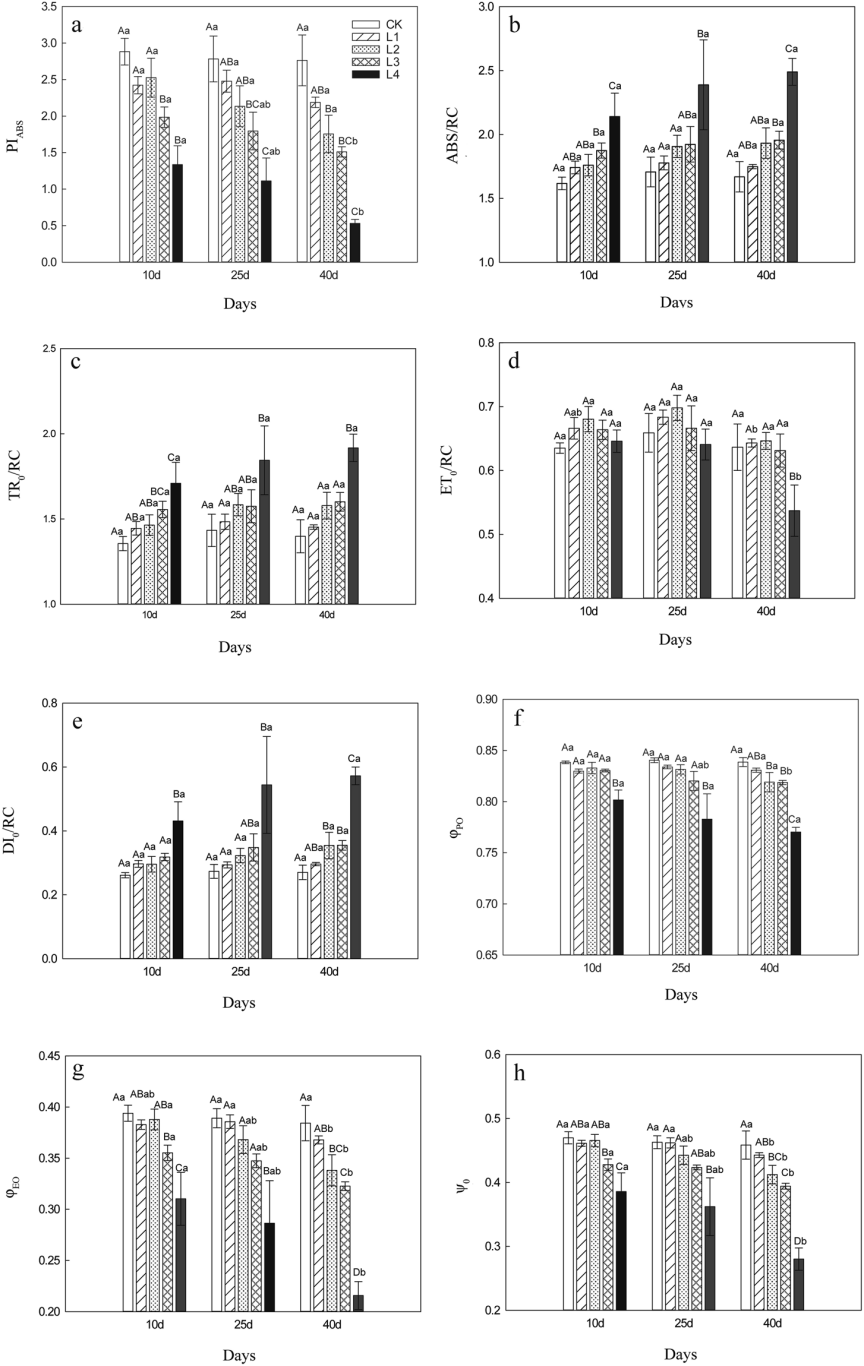
Mn induced changes in performance index (PI_ABS_, **a**), absorption (ABS/RC, **b**), trapping (TR_o_/RC, **c**), electron transport (ET_o_/RC, **d**), dissipation (DI_o_/RC, **e**), the maximum quantum yield of primary photochemistry (φ_Po_, **f**), the quantum yield of electron transport (φ_Eo_, **g**) and the efficiency (ψ_o_, **h**). Values represent mean ± SE. Different capital letters represent significant difference between the different Mn-treatments in the same stress time points, and different lowercase letters represent significant difference between the same Mn- treatments in the different stress time points (*P* < 0.05).

We analyzed several functional parameters from the JIP-test to further characterize the effect of Mn on PSII of *L*. *lucidum*. Performance index (PI_ABS_) showed a declining trend during the test period (Fig. 3a). PI_ABS_ in the L4-treated group was significantly decreased compared with the L1-treated group and the control group in a time-dependent manner. There was no significant difference between the L1- or L2-treated groups and the control group at any time point, except between the L2-treated group and the control group on day 40. In L3- and L4-treated groups, PI_ABS_ was significantly decreased on day 40 compared with day 10.

Absorption ABS/RC (Fig. 3b), trapping TR_o_/RC (Fig. 3c), and dissipation DI_o_/RC (Fig. 3e) were increased in a Mn-concentration dependent manner during the test period, and these parameters were significantly increased in the L4-treated group compared with the control group during the test period. No significant difference at the same levels of Mn was observed among various time points (day 10, 25 and 40). Electron transport ET_o_/RC (Fig. 3d) first increased and then decreased along with the increase of Mn levels, and the maximum value of ET_o_/RC was observed in the L2-treated group. There was no significant difference between the Mn-treated groups and control group at various time points, except between the L4-treated group and the control group on day 40.

Maximum quantum yield φ_Po_ (Fig. 3f), the probability that an absorbed photon moves an electron further than Q_A_^−^ (φ_Eo_ψ_o_) (Fig. 3g), and the probability that a trapped exciton moves an electron further than Q_A_^−^ (ψ_o_) (Fig. 3h) at various time points showed a declining trend with increasing Mn levels. φ_Po_ was significantly decreased in the L4-treated group compared with the control on days 10, 25, and 40, and φ_Po_ was significantly decreased in L2, L3, and L4-treated groups compared with the control on day 40. φ_Eo_ and ψ_o_ were significantly decreased in the L2-, L3- and L4-treated groups compared with the control on day 40.

### Interaction of stress time and Mn levels for Chl a fluorescence parameters (FPs) in *L*. *lucidum* leaves

All JIP-test parameters significantly varied with the Mn levels, and all parameters, except V_I_, ABS/RC, TR_o_/RC, DI_o_/RC, and φ_Po_, were significantly affected by the stress time. However, none of the parameters significantly responded to the interaction between Mn levels and stress time.

### Effects of Mn on net photosynthesis rate (Pn) and intercellular CO_2_ concentration (C_i_) in *L*. *lucidum* leaves

As reported in Table 4, Pn was significantly decreased in L2-, L3-, and L4-treated groups compared with the control. Further, Pn in the L3- and L4-treated groups was significantly decreased compared with the L1-treated group. C_i_ increased with the Mn levels (Table 4), but no significant differences were observed between the control and the Mn-treated groups.

**Table 4:** Net photosynthesis rate (Pn, μmolCO_2_·m^−2^·s^−1^) and intercellular CO_2_ concentration (Ci, μmol mol^−1^) in the control and Mn-treated groups on day 40.

Mn-treatment	Ck	L1	L2	L3	L4
Pn	5.330 ± 0.337 A	4.563 ± 0.777 AB	3.364 ± 0.492 BC	2.419 ± 0.459 C	1.922 ± 0.289 C
Ci	145.741 ± 12.414 A	154.09 ± 7.245 A	159.422 ± 15.331 A	164.086 ± 22.819 A	193.997 ± 0.043 A

Values represent mean ± SE. Different capital letters represent significant difference among Mn-treatments (*P* < 0.05).

Mn-treatment = Manganese-treatment; Pn = net photosynthesis rate; CK = control; L1 = 12 mM manganese-treatment; L2 = 24 mM manganese -treatment; L3 = 36 mM manganese -treatment; L4 = 48 mM manganese–treatment.

### Total contents of Mn in *L*. *lucidum* leaves, stems, and roots

The total Mn contents of plant organs were significantly higher in the Mn-treated *L*. *lucidum* than in the control except for the Mn levels in the leaves of the L1-treated group (Table 5). The Mn concentrations were highest in the roots, followed by the leaves, and the stems. The Mn content was significantly different among roots, stems, and leaves, except for between the stems and leaves in the L1-treated group.

**Table 5:** Mn content in roots, stems and leaves on day 40.

Mn-treatment	Mn (mg/kg DW)		
	roots	stems	leaves
Ck	168.09 ± 17.15Aa	74.92 ± 0.61Ab	192.85 ± 6.88Aa
L1	1576.16 ± 32.01Ba	827.22 ± 17.48Bb	1014.11 ± 202.98Ab
L2	2427.31 ± 15.60Ba	1015.68 ± 47.06BCb	2069.53 ± 46.66Bc
L3	5183.54 ± 258.48Ca	1186.83 ± 68.92Cb	2788.06 ± 480.70Bc
L4	10990.59 ± 668.02Da	1608.17 ± 121.03Db	5108.64 ± 364.31Cc

Values represent mean ± SE. Different capital letters represent significant difference among Mn-treatments (*P* < 0.05), different lowercase letters represent significant difference in the different plant organs between the same Mn- treatments (*P* < 0.05).

Mn-treatment = Manganese-treatment; DW = dry weight; CK = control; L1 = 12 mM manganese-treatment; L2 = 24 mM manganese -treatment; L3 = 36 mM manganese -treatment; L4 = 48 mM manganese-treatment.

## Discussion

In this study, the OJIP curve was observed to be O-L-K-J-I-P when the Mn levels increased (Figs 1 and 2). The OJIP curve is very sensitive to environmental stress^16,21,24^. In leaves that have been exposed to a disturbed environment for a short period of time, Chl a fluorescence shows a polyphasic rise before J step, and the O-J-I-P becomes O-K-J-I-P and even O-L-K-J-I-P^16,26^.

The L-band (~150 μs) is an indicator of energetic connectivity of the antennae to PSII units^24,27^, implying better excitation energy utilization and system stability of PSII units^21,27^. In our study, the presence of positive L-band in the Mn-treated leaves indicated an inferior performance of antennae connectivity compared to the control leaves and might be a sign of disturbed energy transfer^28^. According to the Grouping Concept and JIP-test^21,26^, the positive L-band implies that the PSII units were less tightly grouped, or that less energy was exchanged between the independent PSII units. Therefore, PSII units of Mn-treated leaves had lower stability and became more fragile. However, an amplitude change in the L-band (from positive to negative) of the L1-treated group was observed from day 10 to day 40 (Fig. 2a,c,e) suggesting that the PSII units had better excitation energy utilization and system stability on day 40 without any irreversible damage. This may be associated with a lack of significant Mn accumulation in the leaves of the L1-treated groups (compared to controls, Table 5).

The K-band can be explained by the imbalance of electron flow from the donor side to the acceptor side in the PSII RCs^28^. When the electron transfer from the OEC to tyrosine Z (Y_z_) is slower than the electron transfer from P680 to Q_A_ and beyond, there is a high accumulation of Y_z_^+^^25^. Thus, this accumulation of Y_z_^+^ causes the appearance of K-step, which is directly associated with an inactivation of the OEC^25^. In this study, the appearance of K step suggested that Mn inhibit the electron flow from the donor to the acceptor side of PSII even at low levels (L1) (Fig. 1a–c). Meanwhile, the presence of positive K-band in the Mn-treated leaves indicates an inactivation of the OEC^24,25^ (Fig. 2d–f). Therefore, it may be inferred that the competition between Ca^2+^ and Mn^2+^^29^ in the OEC led to more sites held by Mn^2+^ in the OEC, and this may depend on the similar ion radius and charge properties of Mn^2+^ and Ca^2+^ ^30^.

OJIP transients can be used to examine the electron transport flux from PSII RCs to PSI through Q_A_ and Q_B_. In this study, leaves in the L3- and L4-treated groups had significantly increased V_J_ compared with the control leaves (Table 2), indicating that high levels of Mn induced the accumulation of Q_A_^−^. This result is consistent with the previous findings^16,26,27^. The increased value of V_I_ could be related to the blockade of electron transport downstream of Q_A_ by Mn stress^31^. This finding is also supported by the decrease of φ_Eo_ and ψ_o_ (Fig. 3g,h), as Q_B_ was unable to be reduced by Q_B_-non-reducing PSII RCs^27,32^. Correspondingly, the higher levels of Q_B_-non-reducing centers blocked electron transport towards PSI^32^. Lower redox state of Q_B_ implies altered reduction potential of PSII at the acceptor side in Mn-stressed plants^17^. Since Q_A_ is in quasi-equilibrium with Q_B_ and the PQ pool, the lower redox potential of Q_B_ will decrease the probability of forward electron transfer between the two quinone acceptors by shifting the redox equilibrium between Q_A_^−^Q_B_ and Q_A_Q_B_^−^ towards Q_A_^−^Q_B_^33,34^.

The significant reduction of PI_ABS_, which is a very sensitive indicator of plant functionality^27^, indicates that excessive Mn may down-regulate PSII function, resulting in prolonged negative effect with irreversible damage. An increase in both ABS/RC and TR_o_/RC, and a decrease in φ_Po_ indicates inactivation of a certain part of RCs, which was most likely due to inactivation of OEC as well as the transformation of active RCs to silent ones, because the functional antenna that supplies excitation energy to active RCs was increased in size^24,27^. However, an increase in ET_o_/RC under low levels of Mn (L1 and L2) implies that these inactive RCs^35^ could prevent further damage to themselves and protect neighboring active RCs in response to the absorbed light energy in the active RCs^36^. Significantly increased DI_o_/RC and decreased ET_o_/RC in the highest Mn treatment group (L4) shows that the excess excitation energy was mostly dissipated^21,24^.

ANOVA results revealed that all JIP-test parameters used in this study were significantly affected by Mn stress (P < 0.05), but the interactive influences of Mn stress and stress time on the examined parameters were not significant (P > 0.05) (Table 3). We also found that *L*. *lucidum* leaves were more sensitive to the Mn levels compared with the stress time. Additionally, ET_o_/RC, φ_Eo,_ and ψ_o_ were significantly influenced by Mn stress time, indicating that the blockage of PSII electron flow beyond Q_A_^−^ was more severe in response to the increasing stress time. The blockage of PSII electron flow was also supported by the phenomena of the accumulation of Q_A_^−^ and the increase in V_J_.

The Mn-induced changes in the shape of OJIP transient curves and other related parameters of *L*. *lucidum* as observed in this study were also found in the studies of Mn-treated *Citrus grandis* seedlings^16^, Al-treated *Citrus grandis*^26^, and Cd-treated *Solanum lycopersicum*^37^. But different from our results here, Cr-treated *Spirodela polyrhiza* was found to have a decreasing trend of TR_o_/RC, indicating that the Cr damages LHCs^38^. Therefore, the sensitivity of different parts of the PSII units vary, and this response is the different for different heavy metals and is species-dependent.

This study found that Pn of the plants in L2, L3 and L4 treatments was significantly lower than that in the control (Table 4), and Pn and Ci were negatively correlated. Therefore the reduced Pn observed in our study was not caused by Ci limitation^39,40^. A negative correlation between Ci and Pn was suggested as an indicator to describe the decrease in carboxylation efficiency by Rouhi *et al*.^41^. A positive relationship between maximum quantum yield of PSII (Fv/Fm) and Pn was also found by Tezara *et al*.^42^. These results suggested that the reduction of Pn could be explained by the limitation in photochemical activity of PSII, which impeded the utilization of CO_2_ in the assimilation process. The current study found that excessive Mn impaired the functional PSII, as supported by the observed positive L-band and the observed decrease in PI_ABS_. Thus, Mn toxicity contributed to the observed significant reduction of Pn through its effects on photosynthetic apparatus^43^.

## Conclusions

We conclude that an excess level of Mn affected the net photosynthesis rate, the OJIP transient, and other related parameters of *L*. *lucidum* seedlings. The imaging of JIP-Test parameters revealed Mn-induced photo-damage on the PSII RCs, including a decrease in energy absorption and excitation energy trapping, and an increase in energy dissipation. The disturbance of the PSII electron transport from the donor side to the acceptor side might be associated with inactivation of OEC. This, in turn, resulted in a decrease in the rate of electron transport beyond Q_A_ and an accumulation of Q_A_^−^.
